# Long-term SARS-CoV-2 Asymptomatic Carriage in an Immunocompromised Host: Clinical, Immunological, and Virological Implications

**DOI:** 10.1007/s10875-022-01313-6

**Published:** 2022-07-02

**Authors:** Michele Spinicci, Alessio Mazzoni, Marco Coppi, Alberto Antonelli, Lorenzo Salvati, Laura Maggi, Gregorio Basile, Lucia Graziani, Nicoletta Di Lauria, Vincenzo Di Pilato, Seble Tekle Kiros, Enrico Beccastrini, Riccardo Saccardi, Manuela Angileri, Michele Cecchi, Maria Grazia Cusi, Gian Maria Rossolini, Francesco Annunziato, Alessandro Bartoloni, Paola Parronchi

**Affiliations:** 1 Department of Experimental and Clinical Medicine, University of Florence, Largo Brambilla 3, 50134 Florence, Italy; 2 Infectious and Tropical Diseases Unit, Careggi University Hospital, Florence, Italy; 3 Department of Surgical Sciences and Integrated Diagnostics (DISC), University of Genoa, Genoa, Italy; 4 Department of Cellular Therapies and Transfusion Medicine, Careggi University Hospital, Florence, Italy; 5 Pharmacy AD Preparation Unit, Careggi University Hospital, Florence, Italy; 6 Department of Medical Biotechnologies, University of Siena, Siena, Italy; 7 Microbiology and Virology Unit, Careggi University Hospital, Florence, Italy; 8 Flow Cytometry Diagnostic Center and Immunotherapy (CDCI), Careggi University Hospital, Florence, Italy; 9 Immunology and Cell Therapy Unit, Careggi University Hospital, Florence, Italy

**Keywords:** COVID-19, Monoclonal, Latent, Persistent, Mutation, Viral microevolution

## Abstract

**Purpose:**

SARS-CoV-2 infection in immunocompromised hosts is challenging, and prolonged viral shedding can be a common complication in these patients. We describe the clinical, immunological, and virological course of a patient with eosinophilic granulomatosis with polyangiitis, who developed the status of long-term asymptomatic SARS-CoV-2 carrier for more than 7 months.

**Methods:**

Over the study period, the patient underwent 20 RT-PCR tests for SARS-CoV-2 detection on nasopharyngeal swabs. In addition, viral cultures and genetic investigation of SARS-CoV-2 were performed. As for immunological assessment, serological and specific T-cell testing was provided at different time points.

**Results:**

Despite the patient showing a deep drug-induced B and T adaptive immunity impairment, he did not experience COVID-19 progression to severe complications, and the infection remained asymptomatic during the follow-up period, but he was not able to achieve viral clearance for more than 7 months. The infection was finally cleared by SARS-CoV-2-specific monoclonal antibody treatment, after that remdesivir and convalescent plasma failed in this scope. The genetic investigations evidenced that the infection was sustained by multiple viral subpopulations that had apparently evolved intra-host during the infection.

**Conclusion:**

Our case suggests that people with highly impaired B- and T-cell adaptive immunity can prevent COVID-19 progression to severe complications, but they may not be able to clear SARS-CoV-2 infection. Immunocompromised hosts with a long-term infection may play a role in the emergence of viral variants.

**Supplementary Information:**

The online version contains supplementary material available at 10.1007/s10875-022-01313-6.

## Introduction

Coronavirus disease 2019 (COVID-19) is challenging in immunocompromised patients, including those living with immune-mediated inflammatory diseases (IMIDs) [1]. Underlying immune system dysfunction and the common use of immunosuppressant drugs can affect the clinical and immunological course of severe acute respiratory syndrome coronavirus 2 (SARS-CoV-2) infection [2]. Whether IMID and other immunocompromised patients are at increased risk of severe COVID-19 form and of death or they otherwise experience a tempered detrimental COVID-related inflammatory responses remains poorly understood. On the other hand, prolonged SARS-CoV-2 shedding is common in immunosuppressed individuals [3–5]. Persistent COVID-19 in immunocompromised patients can span from asymptomatic to severe disease, and recurrent symptoms can last for months [3–7]. Beyond limitations that affect patients’ daily life, chronic SARS-CoV-2 infection in immunocompromised patients has raised concern since this condition has been recognized as potential drivers of intra-host viral evolution, especially after exposure to convalescent plasma [8, 9]. Here, we describe the clinical, immunological and virological course of a patient with eosinophilic granulomatosis with polyangiitis (EGPA), receiving combined T- and B-cell immunosuppressant treatment, who developed the status of long-term asymptomatic SARS-CoV-2 carrier, cleared by SARS-CoV-2-specific monoclonal antibody (mAb) treatment.

## Methods

Data were obtained from electronic health records. The study was performed in accordance with the ethical principles of the Declaration of Helsinki and with the International Conference on Harmonization Good Clinical Practice guidelines. Patient’s consent was obtained.

### Viral Culture

Briefly, a 200-μL sample was inoculated into a Vero E6 (VERO C1008 (Vero 76, clone E6, Vero E6); ATCC® CRL-1586™) confluent 24-well microplate for virus isolation. After 1 h incubation at 37 °C in 5% CO_2_, the inoculum was discarded, and 1 mL of medium was added to the well. Cells were incubated at 37 °C and observed with a light microscope every day for a cytopathic effect. After incubation for 7 days, cells were analyzed for the presence of SARS-CoV-2 N protein by using specific antibodies in immunofluorescence assay [10].

### Genetic Investigation

A genetic investigation of SARS-CoV-2 was performed by using EasySeq RC-PCR SARS-CoV2 Whole Genome Sequencing kit (NimaGen BV, Nijmegen, NL) and a MiSeq platform (Illumina, San Diego, US). Sequence analysis was performed by using the virSEAK analysis tool (JSI medical systems GmbH, Ettenheim, DE) to obtain nearly complete SARS-CoV-2 genome sequences, which were deposited in GISAID database (available at www.gisaid.org, EPI_ISL_6630802, EPI_ISL_6630809 and EPI_ISL_6630815).

### Serological Assessment

Presence of neutralizing antibodies was evaluated on Vero E6 cells in a 96-well microplate, using the wild-type SARS-CoV-2/human/ITA/Siena-1/2020 strain (GenBank: MT531537.2), as previously described [11]. The 50% end point geometric mean titer (GMT) was calculated by using the Reed–Muench method [12].

### Evaluation of SARS-CoV-2-SpecificT Cells

For T cell stimulation in vitro, 1.5 million PBMCs were cultured in complete RPMI plus 5% human AB serum in 96-well flat-bottom plates in medium alone (background, negative control) or with a pool of Spike, Membrane, and Nucleoprotein SARS-CoV-2 peptide pools (Miltenyi Biotech) at 0.6 µM/peptide. Staphylococcal enterotoxin B SEB 1 µg/mL (Sigma Aldrich) was used as a positive control. After 2 h of incubation at 37 °C, 5% CO_2_, Brefeldin A (5 µg/mL) was added, followed by an additional 4-h incubation period. Finally, cells were fixed and stained by using fluorochrome-conjugated antibodies listed in the Supplemental data. Samples were acquired on a BD LSR II flow cytometer (BD Biosciences).

## Results

In middle March 2020, a 57-year-old Italian man, with a 9-month history of EGPA reported fever, dry cough, anosmia, and headache soon after a household contact with a confirmed COVID-19 case. He did not seek confirmatory test, as commonly occurred in the very first period of the pandemic in Italy, being classified as a probable COVID-19 case. He recovered spontaneously in a few days and resumed his life without limitations. At that time, immunosuppressant therapy for EGPA included low-dose steroids (methylprednisolone 0.15 mg/kg/day) and mycophenolate mofetil (1000 mg per day), and he had received the third 1000 mg rituximab (RTX) infusion in March 2020, 6 days before the probable COVID-19 (the first two infusions were administered 15 days apart, 3 months earlier). The patient had a history of adult-onset asthma and nasal polyps with olfactory impairment. EGPA — diagnosed 9 months earlier due to fever, acute dyspnea, acute kidney injury, and peripheral neuropathy with hypereosinophilia, high serum eosinophilic cationic protein levels, perinuclear-ANCA pattern on indirect immunofluorescence assay, high anti-MPO antibody levels, and increased IgE levels — was in remission and under almost complete symptoms control with only mild paresthesia of the right foot digits.

**Fig. 1:**
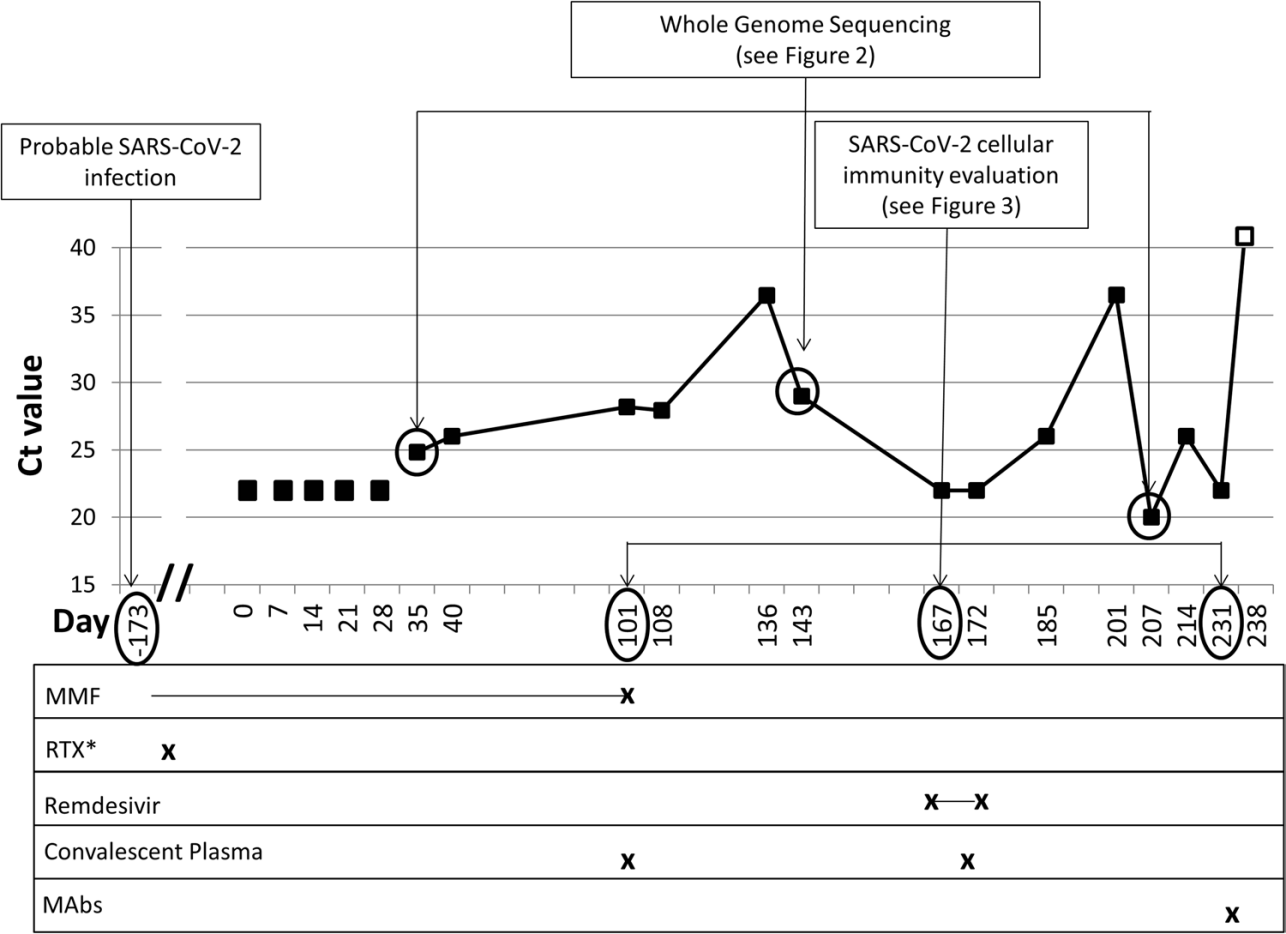
Timeline of diagnostic tests and treatment for SARS-CoV-2. Dots on the graphic represent the cycle threshold (Ct) values of the 19 PCR testing on nasopharyngeal swab performed by the patient between days 0 and 238. Ct values were not available for the first five swabs (days 0–28 in brackets); the last one (day 238) was negative for SARS-CoV-2 (Ct values > 40)

Almost 6 months later (day 0; 173 days after symptoms onset of probable SARS-CoV-2 infection), before acceding to the hospital outpatient service for EGPA visit in total well-being, the patient underwent a screening test for SARS-CoV-2 on nasopharyngeal swab (NS), by Allplex™ 2019-nCoV (Seegene Technologies Inc; Seoul, South Korea) reverse transcriptase–polymerase chain reaction real-time (RT-PCR), which resulted positive. The patient was confined at home, showed no signs or symptoms of COVID-19, and serial follow-up testing was started. Between day 0 and day 231 (> 7 months), SARS-CoV-2 RNA was invariably detected by RT-PCR on a total of 18 consecutive NSs (Fig. 1). Furthermore, on days 40, 108, and 143, SARS-CoV-2 was detected on NS specimens by using in vitro Vero cell cultures, while culture yielded negative on NS specimens collected on days 185 and 214. Due to the persistent SARS-CoV-2 positivity, mycophenolate mofetil was discontinued on day 101, and RTX infusions were suspended after a fourth administration in June 2020. Based on anecdotal reports, two infusions of high-titer (1:640) COVID-19 convalescent plasma (days 101 and 172) and a 5-day course of remdesivir (days 167–172) were provided [13, 14]. In both cases, the SARS-CoV-2 RT-PCR quantification cycle (Cq) value showed a temporary increase soon after the administration, suggesting a partial reduction of the viral load in response to the treatment, although SARS-CoV-2 continued to be detected on NSs and Cq values decreased again within the subsequent weeks (Fig. 1).

Viral clearance was eventually obtained on day 238, 7 days after receiving the SARS-CoV-2-specific mAbs cocktail bamlanivimab 700 mg and etesevimab 1400 mg (Eli-Lilly). The patient remained completely asymptomatic for the whole period, and blood tests performed on days 101, 167, and 231 did not show meaningful abnormalities, including inflammatory biomarkers within normal values. Notably, no secondary cases of COVID-19 were detected among his close contacts, including relatives, work colleagues, and friends, both in the pre- and post-confinement period (i.e., before and after day 0).

**Fig. 2:**
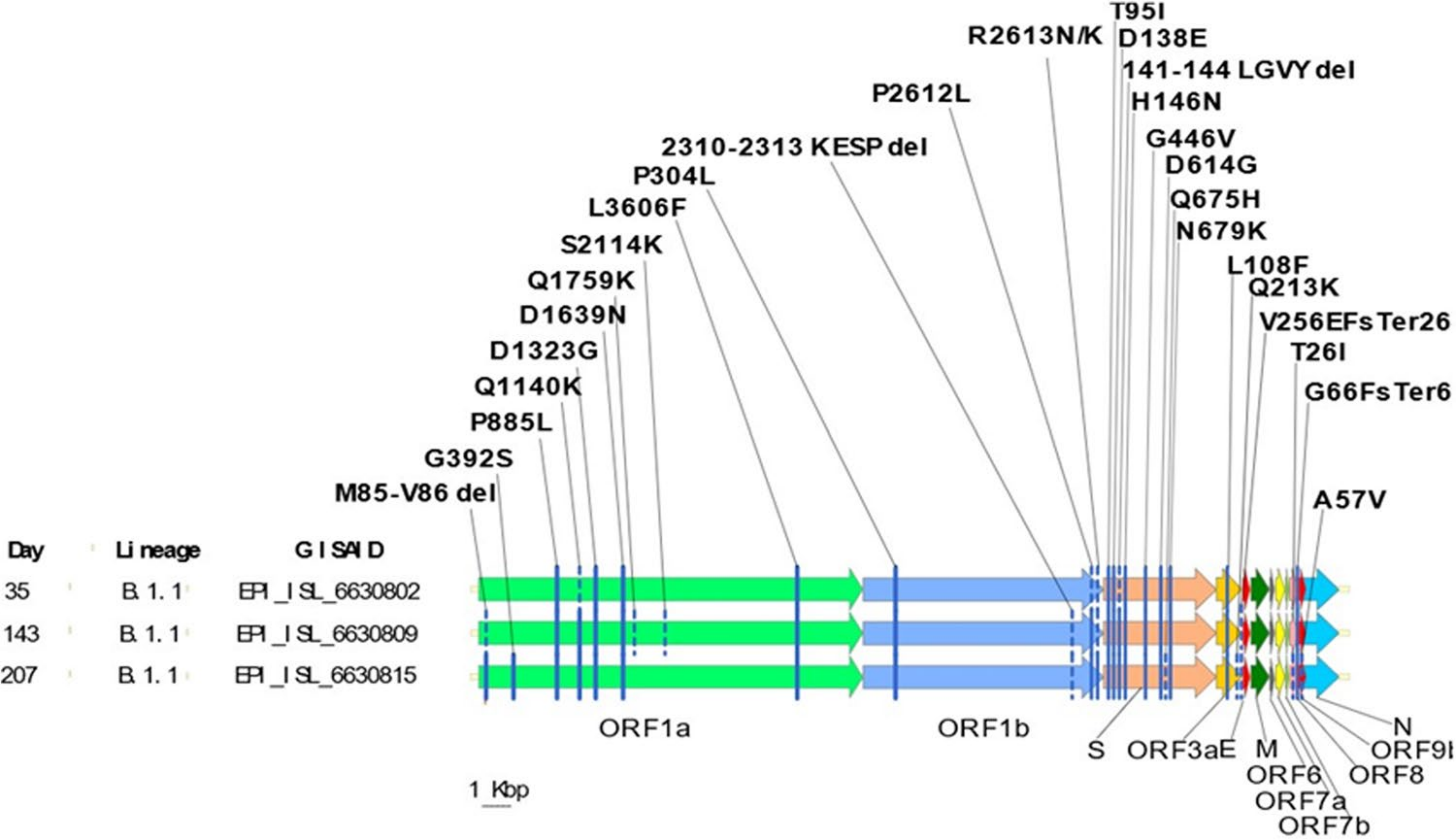
Linear map of SARS-CoV-2 genome with non-synonymous mutations, detected over three different times from an immunocompromised patient with prolonged infection. Mutations constantly detected in all samples are represented by a continuous blue line on the gene, while the dotted line indicates the presence of a mutation occurred with a relative frequency ≤ 90% in that sample

Following the genetic investigation performed on nasopharyngeal samples collected on days 35, 143, and 207, Pangolin v3.1.16 and Nextclade v.1.7.2 assigned all the sequenced samples in the B.1 lineage and 20A clade, respectively, the origin of which is considered linked with the Northern Italian outbreak in February 2020. This lineage is characterized by a synonymous substitution (nt. 3037) in ORF1ab, and by the non-synonymous substitutions P304L (nt. 14408) in ORF1b and D614G (nt. 23403) in the Spike protein (S) [15]. In addition to D614G, known to be associated with a slight increase of transmissibility, several other substitutions were identified in the S locus in all sequenced samples, including (i) T95I, which was reported in several lineages including the variants of concern/interest (VOC/VOI) Delta (B.1.617.2) and Mu (B.1.621), included in vaccine breakthrough infections [16]; (ii) D138E and H146N in the N-terminal domain (NTD), which were reported in Alpha, Beta and Delta variants with an extremely low frequency (≤ 0.001%) according to GISAID (last accessed on April 29, 2022); (iii) G446V, which was located in the receptor-binding domain (RBD) and was related with in vitro decreased sensitivity to convalescent sera [17] and monoclonal antibody REGN10987 [18]; and (iv) N679K, which was described both in the South African C.1.2 and the Brazilian P.1.3 and P.1.4 variants and is located just upstream of the S1/S2 junction, which may enhance Furin cleavage potentially increasing infectivity (Table 1) [19]. Interestingly, while some substitutions in the S protein were constantly detected in all samples within the 7-month time span of infection (e.g., T95I, D138E, H146N, G446V, N679K), several others showed marked heterogeneity in terms of samples where these were identified and of relative frequency (rf) within each sample (Table 1; Fig. 2). As an example, while in the samples of days 143 and 207 a 141–144 LGVY deletion was identified in the S gene with a 98% rf, in the remaining one, it showed an 87% rf (i.e., about 9% of reads with a wild-type sequence were present). This phenomenon could be related to the simultaneous presence of highly similar, but not identical, SARS-CoV-2 subpopulations undergoing micro-evolution during the long-lasting viral carriage.

**Table 1:** Genetic mutations identified in the samples collected at three different times compared with the SARS-CoV-2 reference Genome Wuhan Hu-1 (Acc. No. NC_045512.2)

Genome region	Nucleotide position(s)	Nucleotide change(s)	Amino acid change(s)	Day 35 (09 Oct. 2020)	Day 143 (25 Jan 2021)	Day 207 (30 Mar 2021)
ORF1a	518–523	ATGGTT > –	M85-V86 del	–	+ Sbp (12%)	+
	1439	G > A	G392S	–	–	+ Sbp (16%)
	2919	C > T	P885L	+	+	+
	3683	C > A	Q1140K	+ Sbp (82%)	+	+
	4233	A > G	D1323G	+	+	+
	5180	G > A	D1639N	+	+	+
	5540	C > A	Q1759K	–	+ Sbp (81%)	–
	6606	C > T	S2114K	–	+ Sbp (82%)	–
	11,083	G > T	L3606F	+	+	+
ORF1b	14,408	C > T	P314L	+	+	+
	20,396–20,407	AGGAATCACCTT > ATT	2310–2313 KESP del	–	+ Sbp (8%)	+ Sbp (67%)
	21,302	C > T	P2612L	+ Sbp (77%)	+	+
	21,304–21,306	CGC > AAC	R2613N	+ Sbp (77%)	+ Sbp (85%)	–
		CGC > AAA	R2613K	–	+ Sbp (14%)	+
S	21,846	C > T	T95I	+	+	+
	21,976	T > G	D138E	+	+	+
	21,984–21,995	TTGGGTGTTTAT > –	141–144 LGVY del	+ Sbp (87%)	+	+
	21,998	C > A	H146N	+	+	+
	22,899	G > T	G446V	+	+	+
	23,403	A > G	D614G	+	+	+
	23,587	G > C	Q675H	–	–	+ Sbp (35%)
	23,599	T > A	N679K	+	+	+
ORF3a	25,714	C > T	L108F	+	+	+
	26,029	C > A	Q213K	–	–	+ Sbp (64%)
	26,159–26,160	TT >	V256EFsTer264	–	+ Sbp (88%)	+ Sbp (35%)
ORF8	27,970	C > T	T26I	–	–	+ Sbp (40%)
	28,089–28,093	GGTTC > TGA	G66FsTer66	+	+ Sbp (11%)	+ Sbp (64%)
	28,089–28,097	GGTTCTAAA > TGA	G66FsTer66	–	+ Sbp (89%)	+ Sbp (36%)
ORF9b	28,453	C > T	A57V	–	–	+ Sbp (64%)

– the mutation was not detected, + *Sbp (%)* the mutation was present in a subpopulation of reads with a frequency between 5 and 90% (reported in parenthesis), + the variant occurred with a frequency ≥ 90%

The Q675H substitution was identified in the last sequenced samples only, showing an rf of about 35% (Table 1). This mutation is located near the polybasic cleavage site at the S1/S2 junction and was reported in several SARS-CoV-2 VOI circulating worldwide [19]. Other micro-evolution events were identified for amino acidic substitutions occurring within the ORF1a and ORF1b (Table 1). Among them, the M85-V86 deletion in ORF1a was not identified in the first available sample; its rf constantly increased from 12.5 to 97% in samples collected on days 316 and 380, respectively. To date, the role of 2 or 3 amino-acid deletions has not been clarified, even if the deletion events seem to induce a structural change in a flexible region of the non-structural protein 1 (NSP1 protein) [20]. The R2613N (originating from a 3 nucleotides mutation in ORF1b) showed an rf of 77% in the first available sample and of 85% in the sample collected on day 143, where a second variant was concomitantly detected at the same position, namely R2613K, although with a lower rf (i.e., 14%). R2613K became the only one detectable on day 207. Likewise, the Q1140K and P2612L alterations, which emerged as subpopulations since day 208 (82%), were detected with a frequency > 90% in ORF1a and in ORF1b on day 316, respectively, providing evidence for an additional intra-host micro-evolution event. Overall, the Q1140K substitution showed a low frequency with only 22 genomes deposited in GISAID (GISAID database accessed with virSEAK JSI’s SARS-CoV-2 tool (https://virseak.bio/virus/), last accessed on April 29, 2022), providing further evidence of an intra-host evolution.

As far as immunological response is concerned, serological status evaluated on days 40 and 101 demonstrated the absence of neutralizing antibodies. Further serological assays to detect IgG antibodies against the SARS-CoV-2 Spike protein and nucleoprotein were performed on days 167 and 231 (LIAISON®SARS-CoV-2 S1/S2 IgG, DiaSorin Inc., USA; ARCHITECT®SARS-CoV-2 N IgG Immunoassay, Abbot, USA) with negative results. These findings are in agreement with previous RTX treatment (a fourth 1000-mg infusion was administered in June 2020, and it led to complete B cell depletion, which was still demonstrated on days 167 and 231).

**Fig. 3:**
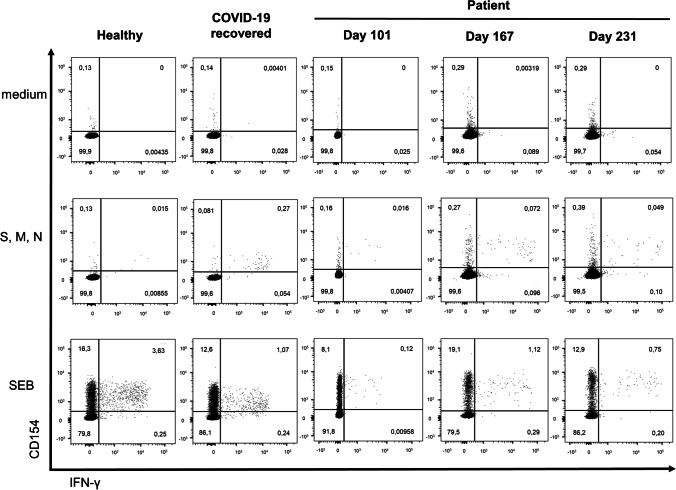
Evaluation of CD4 + T cell response to SARS-CoV-2 antigens or polyclonal stimuli. Flow cytometry plots showing CD154 expression and interferon (IFN)-γ production by CD4 + T cells following SARS-CoV-2 spike (S), membrane (M), and nucleoprotein (N) or SEB stimulation in one healthy non-infected individual, one COVID-19 recovered subject, and in the patient at 3 different time points

Cellular immunity to SARS-CoV-2, assessed on day 101 while the patient was under MMF 1000 mg/day and low-dose steroids (MPDN 0.025–0.05 mg/kg/day), was also significantly impaired. Indeed, the patient displayed absence of circulating CD4 + T cells specific for a pool of peptides spanning the spike, membrane, and nucleoprotein proteins, as compared to a non-infected individual and a subject with history of paucisymptomatic COVID-19 (Fig. 3). It should be noted that the patient’s CD4 + T cells also displayed a significantly impaired cytokine production when stimulated with a superantigen like Staphylococcus enterotoxin B (SEB), thus confirming a profound drug-induced immune suppression. Considering optimal EGPA disease control, MMF was suspended. Drug discontinuation led to a partial recovery of T cell functionality. Indeed, when evaluated on days 167 and 231, respectively 2 months and 4 months after MMF discontinuation, circulating CD4 + T cells showed increased ability to produce cytokines following stimulation with SEB. Moreover, we observed an increase in the frequency of SARS-CoV-2 reactive CD4 + T cells (Fig. 3).

As a proxy for innate immune response, potentially triggered by persistent viral replication, we retrospectively collected measurements of inflammatory parameters on days 101, 167, and 231. According to the asymptomatic status of COVID-19 in the patient, values of interleukin (IL)-6, IL-8, ferritin, and C-reactive protein were in the normal range.

## Discussion

The present case underscores the possibility for a condition of long-lasting SARS-CoV-2 carriage, characterized by absence of clinical symptoms and possibly low infectiousness, in immunocompromised hosts who recover from acute COVID-19. Shedding of infectious SARS-CoV-2 was documented for 143 days, while genomic and subgenomic RNA was detected up to 231 days (> 7 months) after the first positive PCR, but it was reasonable that the infection dated back to March 2020 when probable COVID-19 diagnosis was made (> 13 months). To the best of our knowledge, it is one of the longest SARS-CoV-2 persistent asymptomatic infections reported to date. Although few reports of year-long SARS-CoV-2 detection in immunocompromised hosts have been described, it is unusual to observe a fully asymptomatic patient carrying the virus for more than 6 months [21, 22].

The patient had severe impairment of the adaptive immunity, due to the ongoing immunosuppressant therapies that included RTX, low-dose steroids, and mycophenolate mofetil. B cell-targeting agents, like RTX, profoundly affect B cell functions involved in anti-SARS-CoV-2 immune response [23]. A number of reports have described prolonged or relapsing SARS-CoV-2 infection in patients treated with RTX due to underlying hematological malignancies or IMID [24]. In several cases, despite complete B cell depletion induced by RTX, patients fully recovered from SARS-CoV-2 infection, suggesting that a protective immune response against SARS-CoV-2 could be produced even without humoral immunity [5, 25, 26]. Recently, we reported the case of a late presenter AIDS patient with severe T cell depletion who showed moderate COVID-19 symptoms, but experienced prolonged viral shedding, supporting the hypothesis that also an effective T cell response may be dispensable for the control of COVID-19 progression to severe forms, while it may be necessary for SARS-CoV-2 clearance [27]. In agreement, a 10-month course with MMF profoundly impaired T cell response in this patient. However, T cell inhibition did not result in increased severity of COVID-19, while precluding viral clearance.

Viral clearance was obtained only after mAbs cocktail administration, while remdesivir and convalescent plasma failed in this regard. Prolonged (10-day) or repeated courses of remdesivir were used in immunocompromised patients with alternate results [13, 21, 22, 28]. New oral antivirals against SARS-CoV-2 (molnupiravir and nirmatrelvir/ritonavir) are currently available and widely used in acute COVID-19 patients as the drugs have demonstrated high efficacy in reducing disease progression, but data on their use in patients with prolonged SARS-CoV-2 shedding are lacking. Passive immunization proved to be effective in treating COVID-19 patients with genetic or pharmacological B cell depletion [26, 27, 29]. Currently, convalescent plasma is no longer considered among the standard regimens for COVID-19 treatment due to the unfavorable results obtained from clinical trials [30, 31].

Moreover, a strong selection on SARS-CoV-2 has been reported after convalescent plasma therapy in immunosuppressed individuals, leading to the emergence of viral variants with reduced susceptibility to neutralizing antibodies [12, 21, 22, 32]. Interestingly, the presence of mutations was more frequently observed in samples of days 143 and 207 compared to the earliest available one, and it led to the identification of multiple intra-host microevolution pathways considering the 7-month period of infection. Recurrent deletion regions (RDRs), which map in defined antibody epitopes, were described as sites able to accelerate SARS-CoV-2 antigenic evolution [33]. In this specific case, the viral subpopulation with Δ141–144 might have been selected after the convalescent plasma infusion (days 101 and 172), thus replacing the wild-type subpopulation, identified in the first sample. Moreover, the M85-V86 deletion in ORF1a, which emerged since day 143, might play a role in the regulation of cellular mRNA stability and in suppressing host innate immune functions [20].

Conversely, SARS-CoV-2-specific mAbs demonstrated to be effective in preventing COVID-19 progression and hospitalization, especially when administered in the early phase of the infection and in patients at higher risk of poor outcome, such as the immunocompromised ones [34, 35]. In addition, according to our and other reports, mAbs could have a role also in clearing infection of patients with humoral immunity deficiency, which developed persistent SARS-CoV-2 shedding [19, 21]. Considering the identification of multiple intra-host microevolution pathways, as indicated by the presence of spurious mutations (Table 1) and more frequently observed in samples of days 143 and 207, a tailored treatment is crucial not only for the life quality of these patients, but also for countering the tendency of SARS-CoV-2 to rapidly evolve during the prolonged period of infection [19].

## Conclusions

The complex interaction between SARS-CoV-2 and host immunity is far to be fully disclosed. In immunocompromised patients, SARS-COV-2 infections can exhibit a variety of clinical, virological, and immunological courses. Our case suggests that people with highly impaired B- and T-cell adaptive immunity can prevent COVID-19 progression to severe complications, even if they are unable to clear SARS-CoV-2 infection. This condition can result in a long-term status of asymptomatic SARS-CoV-2 carriage, sustained by multiple viral subpopulations that apparently evolved intra-host during infection.

## Supplementary Information

Below is the link to the electronic supplementary material.

Supplementary file1 (DOCX 13 KB)

## Data Availability

Not applicable.
