# Supplementary material for: Long-term SARS-CoV-2 Asymptomatic Carriage in an Immunocompromised Host: Clinical, Immunological, and Virological Implications
Source: J Clin Immunol. 2022 Jul 2;42(7):1371–8. doi: 10.1007/s10875-022-01313-6 (PMC9674720; doi:10.1007/s10875-022-01313-6)
Supplement: Supplementary file 1 — Supplementary file1 (DOCX 13 KB) [file 10875_2022_1313_MOESM1_ESM.docx]

**Supplementary material**

| **Antigen** | **Flurochrome** | **Clone** | **Company** |
| --- | --- | --- | --- |
| TNF-α | FITC | 6401.1111 | BDBioscience |
| CD154 | PE | TRAP1 | BDBioscience |
| CD3 | PerCP | SK7 | BDBioscience |
| CD4 | PE-Cy7 | SK3 | Invitrogen |
| CD8 | Super Bright 600 | SK1 | eBioscience™ |
| IL-2 | APC | MQ1-17H12 | BDBioscience |
| IFN-γ | Pacific Blue | B27 | BioLegend |
| L/D | Fixable Viability Stain 780 |  | BDBioscience |
